# An informational view of accession rarity and allele specificity in germplasm banks for management and conservation

**DOI:** 10.1371/journal.pone.0193346

**Published:** 2018-02-28

**Authors:** M. Humberto Reyes-Valdés, Juan Burgueño, Sukhwinder Singh, Octavio Martínez, Carolina Paola Sansaloni

**Affiliations:** 1 Department of Plant Breeding/Universidad Autónoma Agraria Antonio Narro, Saltillo, Coahuila, Mexico; 2 International Maize and Wheat Improvement Center (CIMMYT), Mexico City, Mexico; 3 Centro de Investigación y Estudios Avanzados/Instituto Politécnico Nacional, Irapuato, Guanajuato, Mexico; United States Department of Agriculture, UNITED STATES

## Abstract

Germplasm banks are growing in their importance, number of accessions and amount of characterization data, with a large emphasis on molecular genetic markers. In this work, we offer an integrated view of accessions and marker data in an information theory framework. The basis of this development is the mutual information between accessions and allele frequencies for molecular marker loci, which can be decomposed in allele specificities, as well as in rarity and divergence of accessions. In this way, formulas are provided to calculate the specificity of the different marker alleles with reference to their distribution across accessions, accession rarity, defined as the weighted average of the specificity of its alleles, and divergence, defined by the Kullback-Leibler formula. Albeit being different measures, it is demonstrated that average rarity and divergence are equal for any collection. These parameters can contribute to the knowledge of the structure of a germplasm collection and to make decisions about the preservation of rare variants. The concepts herein developed served as the basis for a strategy for core subset selection called HCore, implemented in a publicly available R script. As a proof of concept, the mathematical view and tools developed in this research were applied to a large collection of Mexican wheat accessions, widely characterized by SNP markers. The most specific alleles were found to be private of a single accession, and the distribution of this parameter had its highest frequencies at low levels of specificity. Accession rarity and divergence had largely symmetrical distributions, and had a positive, albeit non-strictly linear relationship. Comparison of the HCore approach for core subset selection, with three state-of-the-art methods, showed it to be superior for average divergence and rarity, mean genetic distance and diversity. The proposed approach can be used for knowledge extraction and decision making in germplasm collections of diploid, inbred or outbred species.

## Introduction

Germplasm banks worldwide contain collections, mainly of cultivated plants and their relatives, to preserve and make available to plant breeders, researchers and general users, their reservoirs of genetic diversity. Those collections are valuable resources to meet the challenges possed by the growing human population and climatic change. A favorable trend has been witnessed during the last 40 years, with a remarkable progress in the assembly and conservation of plant genetic resources [1–3]. Also, the objectives of seed banks have been diversified, and their profiles can be classified as assistentialist, productivist and preservationist [3].

The tools of genome research are helping to unleash the potential of germplasm banks, and are becoming first hand tools for their management. The use of polymorphic DNA markers is helping to discover valuable genes for productivity, disease resistance and abiotic stress tolerance in the accessions contained in those reservoirs. Furthermore, those markers are helping to reduce the genetic redundancy among materials, thus helping the optimization of their contents. Molecular technologies have been demonstrated to be efficient in the finding of genes through linkage maps of genetic markers [1] and more recently by genome-wide association studies called GWAS [4]. In fact, the availability of high-throughput sequencing and genotyping has made possible to examine genome-wide patterns of genetic variation, and link them to phenotypic outcomes. Thus, modern gene banks, empowered by molecular technologies, are revolutionizing the way scientists document the genetic identity of their accessions, track genetic groups and their alleles, identify redundancies, and transform the once static collections into active entities [4].

Rare alleles may be lost due to natural and management-originated bottleneck effects [5], and the sampling schemes used to provide the accessions conforming a gene bank. Although the later is an artificial sampling, its effects can be compared to the bottleneck effects due to population shrinking. Furthermore, rare alleles can be unintentionally left out from collection subsets. Although rare alleles are not likely to have a major importance in the conservation of endangered species, they may be important for plant breeding [6]. The qualification of an allele as rare is usually based on its frequency in a reference population, for example if it is less than 0.1. However, there is still another category of alleles: those that may have a high frequency in a reference population, but are unique among a set of populations. They may be present or even fixed in certain populations due to genetic drift or their relationship with fitness in specific environments. Such uniqueness make them prone to be absent in whole collections and their subsets, albeit their potential importance as a source of important traits for plant breeding. One of the paradigms of this research is to have a theoretical body and a working definition to measure uniqueness of alleles. Novel strategies in germplasm management are needed to preserve those alleles, prone to be lost, which can be potentially useful for plant breeding, specially in a scenario of rapid climate change that represents serious treats to the worldwide food production.

The growth of germplasm banks has created management problems. Large collections are unlikely to be dynamical, with periodic testing and regeneration and field evaluations. Furthermore, duplicates occur very often. Genotyping is contributing to seed bank management by detecting duplicates and helping to select core collections, i.e. subsets of the whole collections that best capture their genetic variation. The creation of core collections was proposed by Frankel [7] to establish a subset with minimum similarity between its entries, chosen to represent the diversity of a large collection, with a manageable size.

Core subsets are useful for evaluating a broad range of variation; however, they may not efficiently capture rare traits or alleles that potentially have high potential breeding or adaptive values [4]. Rare accessions with unique alleles may be neglected because of a lack of obvious desirable traits, but which may harbor genetic information worth to be conserved. Their scarcity and possibility of novel mutations should be considered. The adjective “rare” is well-known in the context of its application to plant species, and it is even quantified with rarity parameters in ecology, based on their frequencies [8]. Since an accession forms part of a genetic continuum in a collection, the frequency-based definition of the rarity of a biological species, a rather discrete unit in the tree of life, does not apply to the entries of a seed bank. To the best of our knwledge, only one definition of the rarity of an accession, based on marker data, has been proposed, in the context of application to SSR maize data [9]. It is basically the square euclidean distance between the array of allele frequencies in a given accession, and the average frequencies for the whole collection. The emphasis of this measure is not on the presence of unique alleles, but on the differences in the allele frequencies of an entry relative to the average ones. This is a symmetrical metrics, where positive and negative differences between an accession’s allele frequency and the global one, are equally weighted.

In this work, an information theory view of alleles and accessions is offered, based on the concept of mutual information, measuring the reduction of uncertainty caused by the knowledge of the distribution of marker alleles across a collection. The field of information theory was founded by Claude Shannon, and deals with problems of transmission, storage and recovery of information. It has been successfully used in several genetic endeavors [10–13]. We use this approach to define the specificity of alleles and the rarity and divergence of accessions, based on information of polymorphic DNA markers.

## Methods

### Mathematical formulation

#### Summary

We define the mutual information between marker alleles and accessions as the average amount of uncertainty removed about the identity of an accession, by the identification of a given allele. It is called mutual due to its symmetry, by which it also can be defined as the average amount of uncertainty about the identity of a given allele, removed by the knowledge of the accession that contains it. This measure is dependent on the amount of allelic variation among the members of a collection. The so-defined mutual information equals the average allele specificity, defined in this context as the information gained about an accession’s identity, by the random extraction and identification of the allele. The rarity of an accession is defined as the average specificity of the alleles it contains. Finally, the accession divergence is defined as the Kulback-Leibler information criterion, between the allele frequencies of a given entry and the global frequencies for the collection. Both, average rarity and average divergence, are identical with the mutual information between alleles and accessions.

#### Basic notation

The basic input are allele frequencies for different accessions in a universe defined for a given collection. The members can be either autogamous or cross-pollinated species, with the only restriction of having a diploid-like type of reproduction. Let *N* be the number of accessions in a germplasm collection, and *k* the number of different alleles at a given marker locus. The set of accessions will be denoted by *X*_1_, *X*_2_,…,*X*_*N*_ and the alleles by *M*_1_, *M*_2_,…,*M*_*k*_. The number of alleles is arbitrary for any given locus, thus multiallelic loci are allowed.

If we extract an *M*_*i*_ allele randomly from the collection, the probability that it belongs to the *j* - *th* accession is obtained by the Bayes theorem:

$$P\left[X_{j}|M_{i}\right]=\frac{P\left[M_{i}|X_{j}\right]P\left[X_{j}\right]}{\sum_{r=1}^{N}P\left[M_{i}|X_{r}\right]P\left[X_{r}\right]}$$

If we consider the space of accessions as equiprobable, then the probability of anyone is 1/*N*, and the equation is simplified as follows:

$$P\left[X_{j}|M_{i}\right]=\frac{P\left[M_{i}|X_{j}\right]\frac{1}{N}}{\sum_{r=1}^{N}P\left[M_{i}|X_{r}\right]\frac{1}{N}}=\frac{P[M_{i}|X_{j}]}{\sum_{r=1}^{N}P\left[M_{i}|X_{r}\right]}$$

To simplify the notation, let’s define:

$$p_{ij}=P\left[M_{i}|X_{j}\right],$$ (1)

i.e. the allele frequency of *M*_*i*_ within the accession *X*_*j*_; and:

$$p_{i}=\frac{1}{N}\sum_{j=1}^{N}p_{ij},$$

i.e. the average frequency of the allele *M*_*i*_ across accessions. Then:

$$P\left[X_{j}|M_{i}\right]=\frac{p_{ij}}{Np_{i}}$$

The input data to perform the calculations through the formulas to be described are allele frequencies for each accession. A convenient format for the input data is a table with accessions as columns and alleles as rows, with each locus covering as many rows as alleles.

#### Mutual information and allele specificity

The conditional Shannon entropy [14] or uncertainty about the identity of an accession, given a random extraction of the allele *M*_*i*_ is:

$$H\left(X|M_{i}\right)=-\sum_{j=1}^{N}P\left[X_{j}|M_{i}\right]log_{2}P\left[X_{j}|M_{i}\right],$$

which can be demonstrated to be:

$$H\left(X|M_{i}\right)=-\sum_{j=1}^{N}\frac{p_{ij}}{Np_{i}}log_{2}(\frac{p_{ij}}{p_{i}})+log_{2}\left(N\right)\sum_{j=1}^{N}\frac{p_{ij}}{Np_{i}}$$

Now, since $\sum_{j=1}^{N}p_{ij}=Np_{i}$, we have:

$$H\left(X|M_{i}\right)=log_{2}\left(N\right)-\sum_{j=1}^{N}\frac{p_{ij}}{Np_{i}}log_{2}(\frac{p_{ij}}{p_{i}})$$

Without marker information, the entropy or uncertainty of a given random accession is *log*_2_(*N*). Thus, the amount of information [14] about *X* given that the randomly extracted allele from the collection is *M*_*i*_ becomes:

$$\begin{array}{cc} I(X;M_{i}) & =H\left(X\right)-H\left(X|M_{i}\right)\\ & =log_{2}\left(N\right)-log_{2}\left(N\right)+\sum_{j=1}^{N}\frac{p_{ij}}{Np_{i}}log_{2}(\frac{p_{ij}}{p_{i}}) \end{array}$$

This is the amount of information that a given marker allele carries about the identity of a random accession. By algebraic simplification of the last expression, we define the **specificity of a marker allele** *M*_*i*_ as follows:

$$S_{i}=\sum_{j=1}^{N}\frac{p_{ij}}{Np_{i}}log_{2}(\frac{p_{ij}}{p_{i}}).$$ (2)

It turns out that Eq (2) is equivalent to the information-based gene specificity in transcriptome analysis [11].

#### Rarity of an accession

The marker allele specificity in Eq (2), provides a framework to define the rarity of an accession, with emphasis on the specificity of its alleles. In this sense, we define the **rarity of an accession** as the weighted average of the specificity of its alleles, with the following expression:

$$R_{j}=\sum_{i=1}^{k}p_{ij}S_{i}.$$ (3)

Eq (3) is equivalent to the tissue specialization in transcriptome analysis [11].

For multiple loci data, rarity can be computed through the average of rarities calculated for each locus. The number of alleles can be variable across loci.

#### Divergence of an accession

To measure of the genomic departure of an accession from the whole collection, we apply the **Kullback-Leibler** divergence between the allele frequency distribution of the *j* - *th* accession and the average allele frequency distribution in the whole set of accessions, defining the **divergence of an accession**:

$$D_{j}=\sum_{i=1}^{k}p_{ij}log_{2}(\frac{p_{ij}}{p_{i}})$$ (4)

This parameter indicates the departure of the distribution of allele frequencies in the *j* - th accession, compared with the average allele distribution. In simple terms, the Kullback-Leibler divergence has been considered as a “measure of surprise” [15]. In the context of allele frequencies, divergence gives an extra weight to the unusual alleles at the whole collection level, since small values of *p*_*i*_ will give large values of *log*_2_(*p*_*ij*_/*p*_*i*_). This is a fundamental difference when compared to the use of the euclidean distance, for which only the differences between *p*_*ij*_ and *p*_*i*_ matter.

In the same way as the rarity case, the divergence scores can be averaged across loci for multiple locus data.

#### A global informational interpretation of those parameters

The mutual information [14] between accessions and marker alleles is:

I(X;M)=H(X)-H(X|M) (5)

The following equality can be proved (see S1 Appendix).

$$I\left(X;M\right)=\sum_{i=1}^{k}p_{i}S_{i}=\frac{1}{N}\sum_{j=1}^{N}R_{j}=\frac{1}{N}\sum_{j=1}^{N}D_{j}$$ (6)

Thus, the ensemble of the allele specificity, accession rarity and accession divergence parameters, is strictly related to the amount of mutual information between the entries of a germplasm collection, and the marker alleles used for their characterization. In an extreme case, a non-polymorphic marker will bear no information about the identity of the accessions in a collection, with a zero allele specificity and null contribution to rarity and divergence.

#### Core subset selection

One proposal of this work is to apply the herein developed theory to select core subsets from a germplasm collection. The optimization criteria is average rarity, defined by Eq (3), which in turn is equivalent to maximizing the average divergence, defined by Eq (4) given Eq (6). The rationale is that, by maximizinig those parameters, we obtain a highly diverse core subset with emphasis on the preservation of rare accessions and specific alleles. The use of an optimization algorithm is needed to select a core subset with maximum rarity. The reason is that rarity is a relative measure, defined in the context of set of accessions; therefore, selection of the rarest accessions of the collection will not provide a subset with maximum rarity, because the value of the parameter for the selected accessions will be re-defined in the context of the universe composed by the core subset. Although several maximization algorithms can be used, we use a greedy algorithm as a heuristic approach [16]. The starting point is the rarest accession, and then consecutively accessions are added under the criterion of causing the maximum average of accession divergence in the growing subset, until a predetermined core size is reached. We use *D*_*j*_ in the optimization iterations, because its expression is simpler than *R*_*j*_, and provides faster calculations.

### Example data sets

#### A simple artificial data set

A small artificial data set is presented in Table 1. It comprises four accessions, A1, A2, A3 and A4, marked with three biallelic loci numbered consecutively from 1 to 3. The numbers in the cells are allele frequencies within accessions, corresponding to *p*_*ij*_, defined by Eq 1. A quick inspection reveals that the Allele 1 of Locus 2 is present only in the accession A4, which gives a maximum specificity score for that allele. The Allele 1 of Locus 3 is a candidate for a higher than average specificity, because it is present in two of the four accessions, but only in A4 has a high frequency. Alleles 1 and 2 of Locus 1 seem to have an average specificity, because they are present in two of the four accessions, with a maximum frequency. The Allele 2 of Locus 2 is present, at its maximum frequency, in two of the three accessions, thus being candidate to low specialization. Accession A4 stands up for having a private allele (Allele 1 of Locus 2), and the highest frequency in another allele (Allele 1 of Locus 3). Thus, a high rarity score is expected for this accession. Although it is not easy to be intuitively perceived by visual inspection, the set of allele frequencies for A4 look different from the average frequencies for all accessions, thus being candidate for a high divergence score. The results of the informational analysis of this data set are described in **Results and Discussion**.

**Table 1:** An artificial data set with four accessions, A1 to A4, marked with three biallelic loci.

Marker	Allele	A1	A2	A3	A4
1	1	1.0	0.0	1.0	0.0
1	2	0.0	1.0	0.0	1.0
2	1	0.0	0.0	0.0	1.0
2	2	1.0	1.0	1.0	0.0
3	1	0.5	0.0	0.0	1.0
3	2	0.5	1.0	1.0	0.0

#### Application to a large wheat landrace collection

Landraces introduced into Mexico from Europe for nearly 5 centuries, also known as Creole wheats, are adapted to different areas in terms of climate, altitude and soil characteristics. From these, 9,811 accessions collected during the 1990s are maintained in the CIMMYT wheat germplasm bank in Mexico. From these entries, 8,416 have been characterized by DArTseq technology, with availability of 20,526 quality SNPs, from the CIMMYT Seeds of Discovery initiative [17]. Data are publicly available in the CIMMYT web page [18].

From the genotyped collection, only the 7,986 hexaploid accessions were used for this study. To score all the alleles in the dataset for their specificities, the whole table of 41,052 alleles x 7,986 accessions was used. However, to obtain reliable scores of rarity and divergence, loci were filtered to have a set with the nearly 10% lowest missing data rates, comprising 4,126 alleles (2,063 SNP loci). With the filtered data, a core subset of 800 lines, representing nearly 10% of the collection, and one representing the 20%, were built by a heuristic maximization of average rarity. Four criteria: mean accession divergence, mean Modified Rogers distance [19], Shannon diversity [20, 21] and alle richness, were used to compare these core subsets with other generated by the state-of-the-arte methods called replica exchange Monte Carlo (REMC) [22], parallel mixed replica exchange (MixRep) and MSTRAT [23]. Although the method employed in [17] for a core subset selection from the same wheat collection is not a direct optimization, but a stratified sampling strategy [24], an additional subset of 1,133 lines was selected with the herein proposed method, to make a basic criteria comparison with the published core. Calculations were performed in the R environment for statistical computing [25], by a script developed to calculate the herein proposed parameters and optimization, which is publicly available in the GitHub site https://github.com/mathgenome/SeedBankInfo. This site contains the data sets used through this research. They can also be retrieved from the CIMMYT repository https://data.cimmyt.org/dataset.xhtml?persistentId=hdl:11529/10547952. Computation of allele specificity and accession rarity has been implemented in Bio-R [26].

For this case study, where markers are biallelic and the data set is large, the algorithm proceeds as follows (i) the table of allele frequencies, with alleles as rows and accessions as columns, is reduced to have only one row for each SNP locus with the frequency of one allele, the other one being complementary, (ii) the rarity of each accession is calculated, (iii) the core subset starts with the rarest accession, (iv) each accession is added to the growing core subset, and the average divergence is calculated in the context of that subset, (v) the accession that gives the maximum average divergence to the subset is kept in the growing core, (vi) the process returns to the step iv, until the desired core size is attained. In order to have fast calculations for very large data sets, divergence is calculated exactly as in (4) until the 30th accession is added. After that, ${\hat{p}}_{i}$ is used insted of *p*_*i*_, with ${\hat{p}}_{i}$ being an estimation based on the growing core before adding the accession being tested. To compare with other approaches fore core subset selection we used the Core Hunter 2.0 software [27].

## Results and discussion

### Analysis of the artificial data set

The analysis of the data set in Table 1, through the application of Eq 2 to the allele frequencies *p*_*ij*_ in the cells of the table, and their row averages *p*_*i*_, with *N* = 4, gave the highest specificity to the Allele 1 of Locus 2, with a value of 2. This is the theoretical maximum for this data set, i.e. *log*_2_(*N*). This score of two bits means that identification of that allele leads to unequivocal identification of the accession bearing it among the set of four. Allele 2 of Locus 2 had the minimum specificity among the whole set of alleles, expected by the visual analysis, with a score of 0.415. The second lowest specificity was found for Allele 2 of Locus 3, with a value of 0.478, being present in three of the four accessions, although with a frequency of only 0.5 in one of them. All other alleles had a rather average specificity close to 1. Rarity of accessions, calculated through Eq 3 as the average of allele specificities weighted by allele frequencies, had its maximum for A4. This accession bears a private allele and another one that is present in two accessions, but with a frequency of only 0.5 in one of them, with a rarity score of 1.361, which is not the theoretical maximum. To reach the theoretical maximum, in this case 2, all of its alleles should be private. The remaining three accessions had rarity scores around 0.63. The maximum divergence was also attained by A4, with a score of 1.472. The minimum divergence was attained by A1, with a value of 0.487. Interestingly, this accession did not have the lowest rarity, but the second highest one, with a score of 0.732. In fact, albeit rarities and divergences have equal overall average values, and are positively related, they do not measure exactly the same phenomenon. Whereas divergence measures the departure from global averages, rarity emphasizes the presence of unusual alleles and takes into account not only the global allele frequency averages, but the particular ones across accessions.

### Analysis of the wheat data set

**Fig 1:**
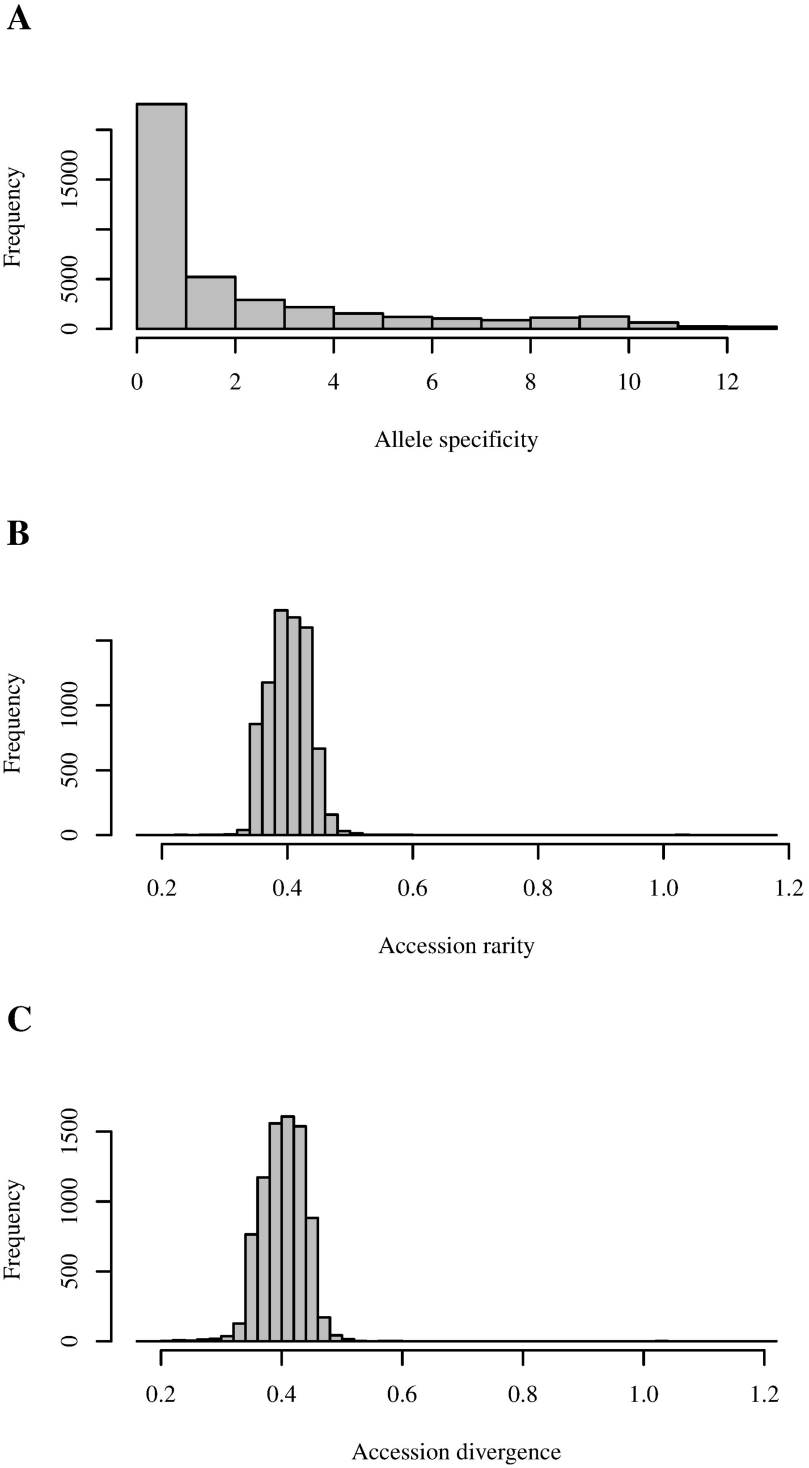
Histograms for the different parameters estimated in the landrace wheat collection. A: Distribution of specificity in 41,052 alleles. B: Distribution of rarity in 7,986 accessions. C: Distribution of divergence in 7,986 accessions.

The average allele specificity from the total of 41,052 alleles had a mean of 2.1695 and a median of 0.7794, showing a right-skewed distribution (Fig 1A), with 55% having values less than 1, and 0.6% having values greater than 12, the maximum theoretical specificity being 12.96326, i.e. the log base 2 of the number of accessions. None of the alleles reached the theoretical maximum, attained when an allele is private of a unique accession; however, three alleles had an specificity of 12.9629. The specificity parameter was successful, because each one of those three alleles was in fact present in only one accession. The theoretical maximum was not attained because of the presence of two missing data points in each one of those three alleles. These alleles were among the cleanest ones, bearing a very low incidence of missing data. The implementation of the specificity estimation has an integrated quality control, in which selection for maximum specificity involves inherently the criteria of searching alleles with a low rate of missing data. The upper bound value of specificity is limited by the number of frequency scores effectively available for the given allele in the data set. This happens because only available values of allele frequency are used, ignoring the cases with missing data. The impact of missing data points on the scores for allele specificity could be reverted using a relative specificity, dividing Eq (3) by the log base 2 of the number of available data for the given marker. However, if some loci are technically susceptible to missing data, we recommend using the absolute specificity to select a reliable set of specific alleles. On the other hand, if the missing data pattern is not random, obeying to missing genome, absolute and relative specificity can be used, with an inspection of their relationship. For the case of alleles that had a specificity of zero, a total of 1,528, they were non-polymorphic, being uniformly present in all accessions. The allele specificity can be valuable to mine rare alleles that could be associated with specific adaptations and has the potential to be used in the screening of accessions for plant breeding.

**Fig 2:**
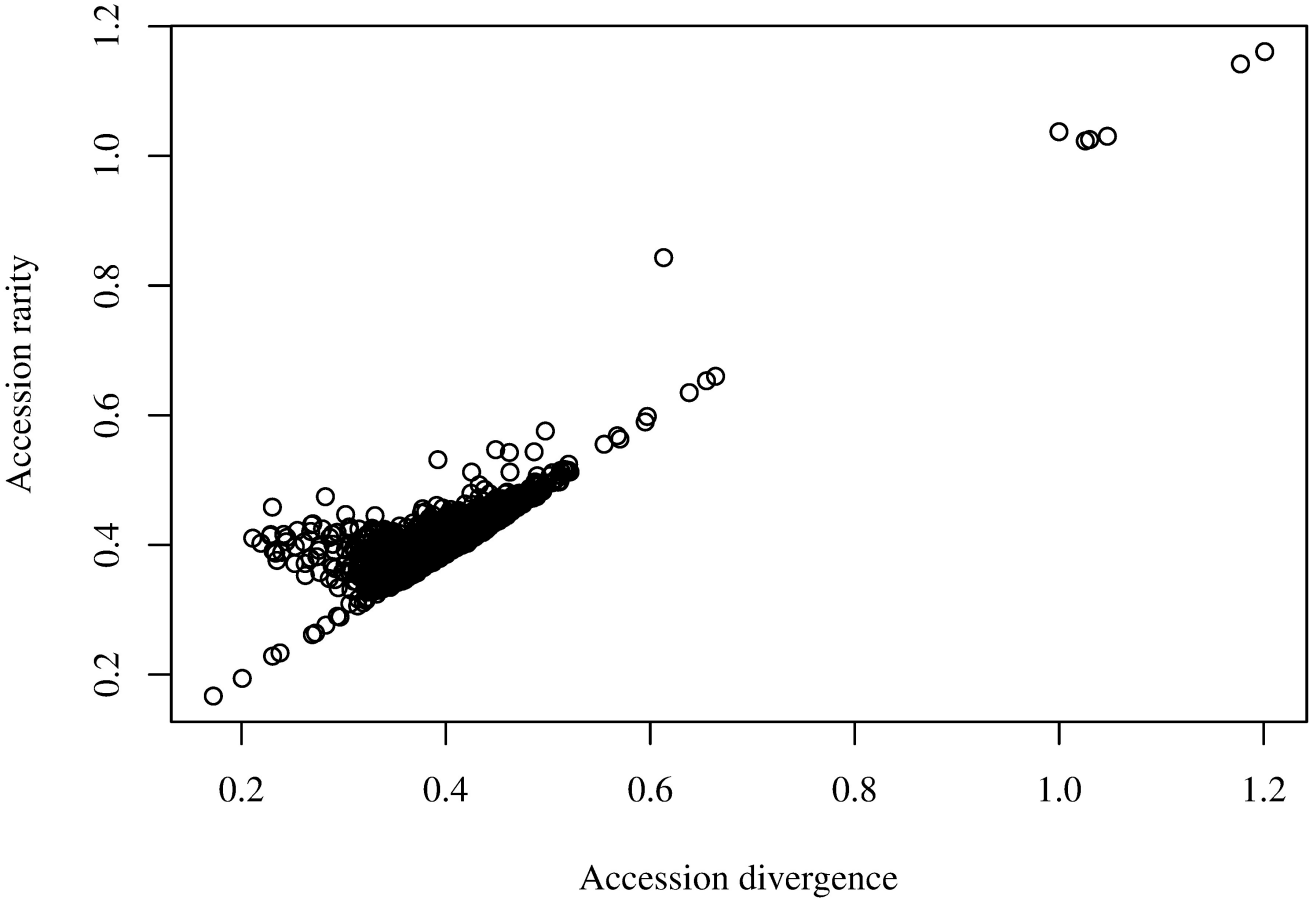
Scatter plot of rarity against diversity in 7,986 wheat accessions.

The rarity of accessions had a mean value of 0.403, with a distribution largely symmetrical, but with extension towards very rare accessions (Fig 1B), with 99.45% of accessions being concentrated in rarity values between 0.3 and 0.5. A group of six accession was found in the right side of the distribution, with values greater than 1. Divergence had a very similar distribution (Fig 1C), with the most divergent accession being the same as the rarest ones. The association between rarity and divergence was mainly linear, but with notorious deviations in which rarity exceeds divergence, attributable to the presence of very specific alleles in those accessions (Fig 2). Nonetheless, as predicted by the theory, the average accession rarity was exactly equal to the average accession divergence. The use of rarity, defined by Eq (3), has a fundamental difference with divergence, defined by Eq (4): while rarity considers the allele distribution across all accessions, divergence is only based on the allele frequencies of the given accession and the average of the collection, which is the same case of the use of the square euclidean distance [9].

**Fig 3:**
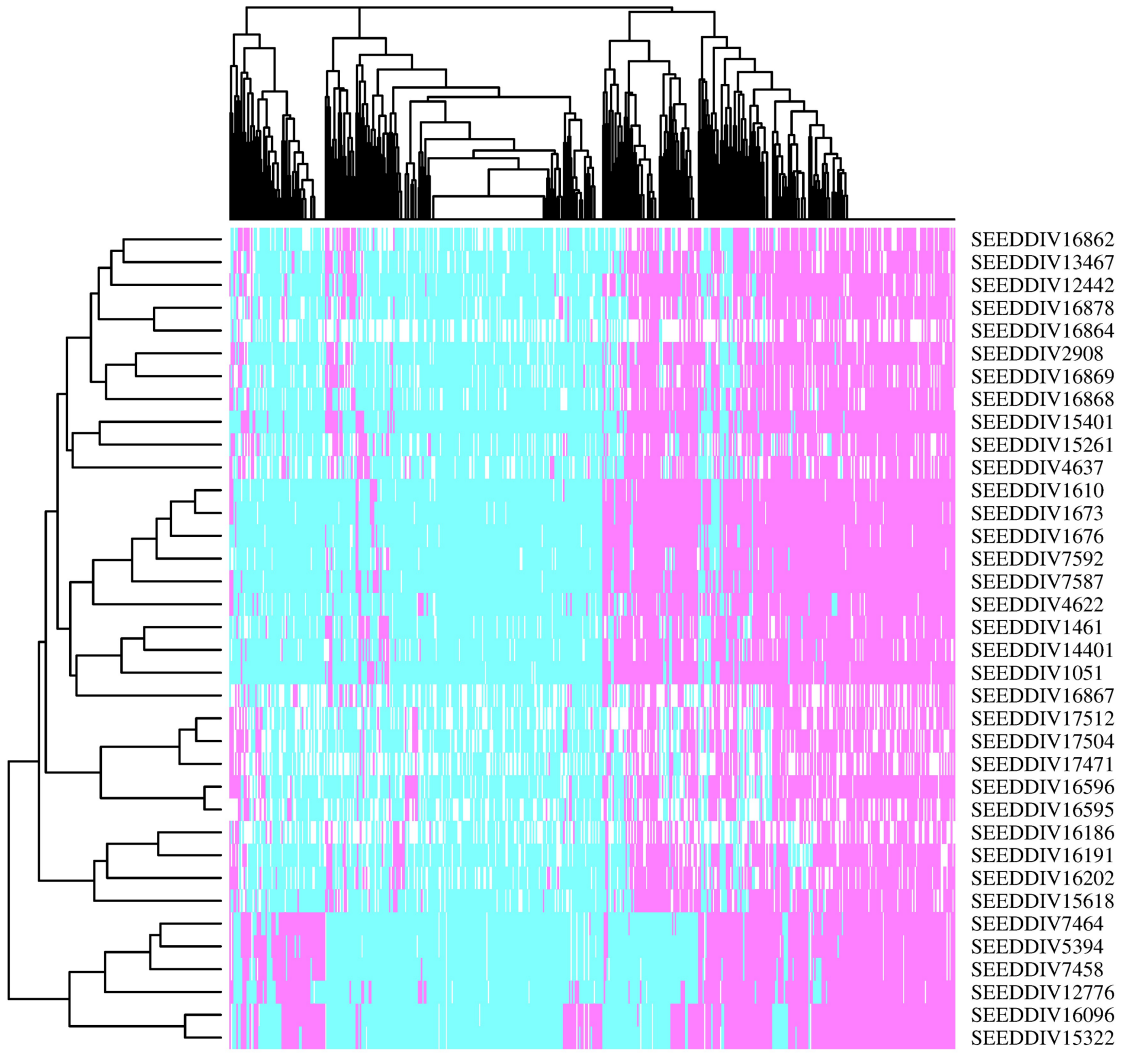
Heat map for 30 common and six rare accessions, genotyped with a sample of 500 alleles. The dendrogram on the X axis represents the 500 alleles, whereas the one on the Y access represents the 36 accessions. Allele absence is shown in turquoise, allele presence in violet, and missing data in white.

In Table 2, the six wheat accession with maximum rarity are listed, along with their rarity and divergence scores, as well as their geographic data. Coincidentally, four of the six accessions were collected in the same region, in the state of Michoacán, while the other two are registered for the states of México and Chihuahua. Fig 3 depicts a heat map, with the six rarest accessions and the 30 less rare, based on random sample of 500 alleles. The six rarest accessions appear at the bottom of the plot, forming a cluster clearly separated of the remaining accessions. The four accessions of Michoacàn appear forming a sub-tree, while the accessions from the México and Chihuahua states appear very close to each other. In fact, both accessions are mutually nearest neighbors in the SNP marker landscape. The same kind of plot was assayed against larger sets of common (less rare) accessions, and the rarest ones still appeared as a single cluster. The rarity parameter in Eq (3) can effectively be used to make decisions about the conservation of a given accession. A set of very rare materials is worth to be preserved, even when a practical use of it is not immediately known, just for the fact that its loss can mean the loss of very rare or specific alleles. A table with all accession names, rarity and divergence is provided in S1 File.

**Table 2:** The six wheat accessions with maximum rarity, along with their scores of Kullback-Leibler divergence based on the information of 4,126 SNP alleles, and geographic locations in Mexico.

Accession	Rarity	Divergence	Region	State	Locality	Longitude	Latitude	Elevation (m)
SEEDDIV16096	1.161	1.201	North	Chihuahua	Jicamorachi	-108.308	27.916	1746
SEEDDIV15322	1.142	1.177	Central	México	Río Frío	-98.833	19.317	2233
SEEDDIV7458	1.037	1.000	Central	Michoacán	La Zarzamora	-101.500	19.183	2200
SEEDDIV5394	1.030	1.047	Central	Michoacán	La Zarzamora	-101.500	19.183	2200
SEEDDIV7464	1.025	1.030	Central	Michoacán	La Zarzamora	-101.500	19.183	2200
SEEDDIV12776	1.023	1.026	Central	Michoacán	Toquara	-101.500	19.183	2000

A core subset of 800 wheat lines was selected by heuristic maximization of the average divergence, which in turns maximizes average rarity. The selected accessions are marked in S1 File. In Table 3 a comparison of methods is presented, based on four criteria: average divergence, mean modified Roger’s distance (MR), Shannon diversity (SH) and allele richness (AR) as the fraction of the number of alleles relative to the whole collection. The number of lost alleles (LA) is derived directly from allele richness and cannot be considered as an additional criterion, but it can be illustrative for comparison. The reference methods are REMC, MixRep, and MSTRAT [23], with a random sample as a comparison control. The method herein proposed for core selection is called HCore, due to the inherent use of Shannon entropy, commonly represented by *H*. Mean Rogers’s distance (MR) and Shannon diversity (SH) were the components of the objective function for the other three methods, with default weights of 70% and 30% respectivelly in the Core Hunter 2.0 software [27]. For average accession divergence, HCore outperformed all other methods, which is not surprising because our maximization was aimed to that parameter. The superiority of the mean divergence over the random sample for HCore is close to 10 times that of the worst performing method: MSTRAT, although only 1.2 times superior to MixRep.

**Table 3:** Comparison between HCore and other three approaches for an approximate 10% core subset selection through several criteria.

Method	Divergence	MR	SH	AR	LA
HCore	0.442	0.438	7.954	0.985	59
MixRep	0.435	0.435	7.950	0.986	53
REMC	0.408	0.419	7.932	0.975	98
MSTRAT	0.406	0.417	7.931	0.983	67
Random	0.402	0.416	7.929	0.976	95

Divergence is the average Kullback-Leibler divergence, MR is the average modified Roger’s distance, SH is the Shannon diversity, AR is the allele richness, as a percentage of the alleles present in the whole collection, and LA (lost alleles) refers to the number of alleles of the whole collection that are not present in the core subset. The last row presents the values of the criteria in a random sample.

For MR, the HCore method outperformed MixRep, REMC and MSTRAT. The same behavior ocurred with SH, where HCore had higher scores than MixRep, REMC and MSTRAT. For allele richness (AR) the best score was for MixRep, followed by HCore. The number of lost alleles in HCore was only six above MixRep. It is notorious that REMC generated a core with more lost alleles than the random sample. The fairly good behavior of HCore for MR, SH and AR, even when they were not components of the objective function, can be considered a collateral effect of maximizing the Kullback-Leibler divergence. Very small differences in MR, SH and AR are typically observedl when comparing core selection methods [27]. On the other hand, the composition of different cores subsets can be more contrasting than the small differences between criteria may suggest.

An additional test result is presented in Table 4, with a 20% core subset selection. HCore had the same comparative behavior as with the approximate 10% core subset. It outperformed the remaining four approaches in Divergence, MR and SH, ranking in second place for AR, after MixRep. The only differences occurred in the ranking among the remaining four approaches. For Divergence, MR and SH, random selection outperformed REMC and MSTRAT. For AR, the ranking was the same as with the 10% core subset, with MixRep having the maximum score and random selection the minimum. This additional test reinforces the usefulness of the informational parameters herein proposed, in their application to core subset selection.

**Table 4:** Comparison between HCore and other three approaches for a 20% core subset selection through several criteria.

Method	Divergence	MR	SH	AR	LA
HCore	0.434	0.434	7.948	0.988	46
MixRep	0.428	0.431	7.945	0.990	40
REMC	0.402	0.416	7.929	0.986	53
MSTRAT	0.402	0.416	7.929	0.987	51
Random	0.404	0.417	7.930	0.985	60

The core subset of 1,133 lines generated by HCore with the low missing data genetic markers, had the following values for the evaluated criteria: Divergence, 0.438; MR, 0.436; SH, 7.951; AR, 0.987; and LA, 52. The subset published by [17] had the following scores for the same marker set: Divergence, 0.404; MR, 0.417; SH, 7.930; AR, 0.983; and LA, 66. To do a more objective comparison for allele richnes (AR), an independent calculation was performed by considering the 20,526 loci of the dataset, comprising the 2,063 SNP loci used for optimization with HCore plus the remaining 18,463 loci. With this information, the HCore subset had an AR value of 0.989, whereas the published core had a value of 0.978. Thus, the superiority of allele richness for the subset generated by HCore was preserved. Although the HCore criteria values outperformed those of the published core subset, we must consider that both cores were selected under different criteria. Furthermore, in [17] phenotypic information was employed, and the SNP information was not used directly, but reduced to 2,000 principal components, with the six principal axes of a hierarchical multiple-factor analysis being selected to represent genotypic and phenotypic variances.

Core subsets are selected for diverse objectives, and in the case of our method, it is aimed to conserve the genetic diversity of the collection, while preserving rare accessions and uncommon alleles. Furthermore, as it happens with the other methods, this approach can be used in combination with phenotype based hierarchical clustering, aiming to preserve genetic diversity within groups. On conceptual grounds, the theoretical underpinning of this approach for core subsets is a rather solid one: the subset of accessions with maximum average divergence is the one that has the maximum mutual information between genotypes and accessions.

The herein proposed definitions of specificity of alleles and rareness of accessions, represent an advance in analytical tools to preserve diversity in plants, with special impact on those of interest in agriculture. Common alleles tend to be ubiquitous in crop collections; however, the unique ones are in danger to be neglected and lost. Thus, objective criteria to make decisions for conservation are highly valuable. Rare populations and specific alleles grow in importance for mainly two reasons: climate change and the availability of novel breeding tools. In the first case, despite much uncertainty derived from different assumptions for climate change projections, exhaustive analyses point towards a scenario where food security is clearly threatened by climate change in the near-term [28]. In the second case, useful genetic variants can be mined and used in plant breeding through the available genomic tools, to produce adapted cultivars to face the diverse climate change scenarios and their direct and indirect implications for food production [29].

## Conclusions

We provide a novel view of a collection in a germplasm bank tied to marker data. It can be considered as a digital view, in the sense that genomes are binarily coded through molecular markers, providing the elements for an informational landscape, where allele specificities are defined in terms of their information about the identities of the accessions, and accession rarities are defined by the average specificity of their alleles. Furthermore, it was found that the average rarity in a collection equals the average Kullback-Leibler divergence of each accession from the global allele frequencies of a germplasm collection. This view can be used to make decisions for conservation of genetic diversity, while avoiding to neglect rare variants. Application of this approach to a large collection of wheat landraces allowed us to rank the alleles according to their specificities, detecting those that are largely private of a few accessions. Furthermore, by ranking the accessions by their rarity and divergence, we could detect a group of rare accessions, which may have unique genetic potentials. This informational view, used as a tool for core subset selection in a large wheat collection, produced favorable results, with an ensemble of accessions with higher average Kullback-Leibler divergence, average Modified Roger’s distance and Shannon diversity than those produced by three state-of-the-art methods, and with fairly good results for allele richness. This does not imply that the application of our method to select core subset is better than all others, but it effectively aims to obtain core collections with a high average Kullback-Leibler divergence, with positive collateral effects in other criteria. Although the methods were applied to largely homozygous lines, definitions are based on allele frequencies and can be equally applied to collections of outbred crops or wild species with diploid-like meiotic segregation.

## Supporting information

S1 Appendix Mathematical proof. Equality between mutual information, average rarity and divergence.
(PDF)

S1 File Scored accessions. Accession names, rarity and divergence for all hexaploid lines. The members of the core subset are marked in the last column.
(CSV)
