# Supplementary material for: An informational view of accession rarity and allele specificity in germplasm banks for management and conservation
Source: PLoS One. 2018 Feb 28;13(2):e0193346. doi: 10.1371/journal.pone.0193346 (PMC5831390; doi:10.1371/journal.pone.0193346)
Supplement: S1 Appendix — Equality between mutual information, average rarity and divergence. (PDF) [file pone.0193346.s001.pdf]

S1 appendix. Equality between mutual information and average rarity and divergence

August 23, 2017

For a given locus, the mutual information between accessions (X) and alleles (M) can be defined as follows

$$I(X;M) = H(X) - H(X|M),$$

where $H(X|M)$ is the conditional entropy of X given M. This is equivalent to:

$$I(X;M) = \sum_{i=1}^{k} p_i I(X;M_i)$$

Now, since $I(X;M_i) = S_i$, and considering expression (2), substitution leads to:

$$\begin{aligned} I[X;M] &= \sum_{i=1}^{k} p_i S_i \\ &= \frac{1}{N} \sum_{j=1}^{N} \sum_{i=1}^{k} p_{ij} S_i \\ &= \frac{1}{N} \sum_{j=1}^{N} R_j \end{aligned}$$

$I(X;M)$ can be rewritten by substitution with expression (1)

$$\begin{aligned} I[X;M] &= \sum_{i=1}^{k} p_i S_i \\ &= \sum_{i=1}^{k} p_i \sum_{j=1}^{N} \frac{p_{ij}}{Np_i} log_2 \left(\frac{p_{ij}}{p_i} \right) \\ &= \sum_{i=1}^{k} \frac{p_i}{Np_i} \sum_{j=1}^{N} p_{ij} log_2 \left(\frac{p_{ij}}{p_i} \right) \\ &= \frac{1}{N} \sum_{ji=1}^{N} \sum_{i=1}^{k} p_{ij} log_2 \left(\frac{p_{ij}}{p_i} \right) \\ &= \frac{1}{N} \sum_{j=1}^{N} Dj, \end{aligned}$$

i.e. the average **Kullback-Leibler** divergence, described in expression (3).
In synthesis, this proofs that:

$$\boxed{I[X;M] = \frac{1}{N}\sum_{j=1}^{N} R_j = \frac{1}{N}\sum_{j=1}^{N} D_j,}$$

i.e. the mutual information and average rarity are equal to the average Kullback-Leibler divergence between accessions and the pooled allele frequencies.
